# Kinetics of rabies antibodies as a strategy for canine active immunization

**DOI:** 10.1186/1678-9199-20-37

**Published:** 2014-08-26

**Authors:** Selene Daniela Babboni, Hení Falcão da Costa, Luzia de Fátima Alves Martorelli, Ana Paula de Arruda Geraldes Kataoka, Cassiano Victoria, Carlos Roberto Padovani, José Rafael Modolo

**Affiliations:** 1 Department of Veterinary Hygiene and Public Health, School of Veterinary Medicine and Animal Husbandry, São Paulo State University (UNESP – Univ Estadual Paulista), Botucatu, São Paulo State, Brazil; 2 Department of Animal Health and Production, Veterinary Medicine School, São Paulo State University (UNESP – Univ Estadual Paulista), Araçatuba, São Paulo State, Brazil; 3 Laboratory of Zoonoses and Vector-Borne Diseases, Center for the Control of Zoonosis of São Paulo City, São Paulo, São Paulo state, Brazil; 4 Department of Biostatistics, Botucatu Biosciences Institute, São Paulo State University (UNESP – Univ Estadual Paulista), Botucatu, São Paulo State, Brazil

**Keywords:** Rabies, Dogs, Vaccination, Revaccination, Annual vaccination campaign, Fuenzalida-Palácios

## Abstract

**Background:**

Rabies, a zoonosis found throughout the globe, is caused by a virus of the *Lyssavirus* genus. The disease is transmitted to humans through the inoculation of the virus present in the saliva of infected mammals. Since its prognosis is usually fatal for humans, nationwide public campaigns to vaccinate dogs and cats against rabies aim to break the epidemiological link between the virus and its reservoirs in Brazil.

**Findings:**

During 12 months we evaluated the active immunity of dogs first vaccinated (booster shot at 30 days after first vaccination) against rabies using the Fuenzalida-Palácios modified vaccine in the urban area of Botucatu city, São Pauto state, Brazil. Of the analyzed dogs, 54.7% maintained protective titers (≥0.5 IU/mL) for 360 days after the first vaccination whereas 51.5% during all the study period.

**Conclusions:**

The present results suggest a new vaccination schedule for dogs that have never been vaccinated. In addition to the first dose of vaccine, two others are recommended: the second at 30 days after the first and the third dose at 180 days after the first for the maintenance of protective titers during 12 months.

## Findings

Half of the world population (3.5 billion people) lives in areas where there is an increase of dogs, cats and rodents, and therefore the frequency of zoonoses transmitted by these animals is also augmented [1]. Globally, rabies provokes 40,000 to 70,000 deaths per year and approximately 15 million people receive post-exposure rabies treatment [2-4]. This means that rabies kills one person every minute worldwide [5]. Rabid dogs are responsible for 99% of these deaths and 92% of post-exposure treatments [1]. In Brazil, 140 people died due to rabies between 2001 and 2010, in 40% of these cases the disease was contracted from dogs, in 1.43% from cats, in 53.57% from bats and in 5% of the cases from other animals [6]. The protection of humans against urban rabies is achieved mainly by prophylactic measures applied to dogs and cats, which include vaccines that induce minimum antibody titers (≥0.5 IU/L) [7]. In Brazil, vaccination against rabies in dogs and cats is mandatory [8]. However, in mass vaccination campaigns, numerous animals do not achieve protective antibody titers after vaccinated with Fuenzalida-Palácios modified vaccine (Institute of Technology of Paraná, TECPAR®) [8-10]. The present study aimed to evaluate during 12 months the kinetics of antibodies in dogs that were first vaccinated against rabies.

The study was conducted in 2009 during the annual vaccination campaign against rabies in dogs and cats in the urban area of Botucatu (22° 88’ 83’ S, 48° 44’ 5” W). A confidence interval of 95% and an estimation error of 10% associated with the casual participation of an animal (50% of positive responses for participation) were considered for the determination of the sample size. In Brazil, the first vaccine shot is administered at three months of age and the booster dose should be given 30–45 days after the initial one, with subsequent annual revaccination [11].

The present study involved 576 dogs older than three months of age, which had never been vaccinated against rabies, regardless of breed, age or sex and randomly selected for blood collection. Samples were collected at five moments of the study period. The first blood sample was taken during the annual vaccination campaign against rabies and the others on home visits, by venipuncture of the cephalic vein, saphenous or jugular (with a 3-mL syringe and 30 × 7 mm needle) and placed in sterile test tubes without anticoagulant. The days of blood collection were labeled as follows: 0 (on the day of the first vaccine dose), 1 [30 days after first vaccination (dafv) and the same day as the second dose of vaccine], 2 (60 dafv), 3 (180 dafv) and 4 (360 dafv).

**Figure 1:**
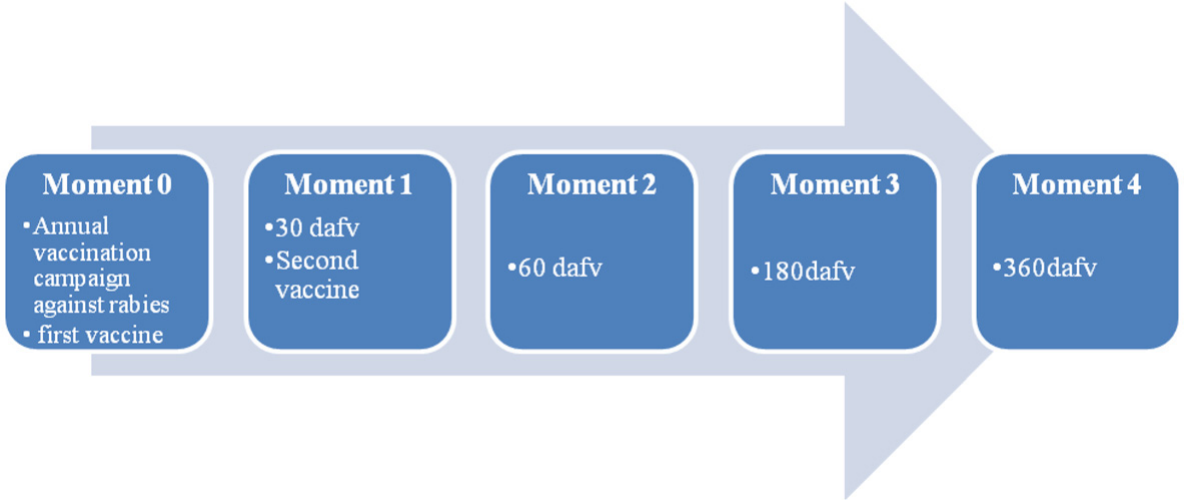
Experimental design of the present study.

Thirty days after the first immunization (moment 1), dogs received the booster dose with the same type of vaccine (Figure 1). This study was characterized as longitudinal and was composed of a single experimental group, since a control group was not allowed (unvaccinated animals in a mass campaign against rabies) according to a city's law [12]. The vaccine employed in this study was from lot number 187/08, each 2-mL dose was subcutaneously injected (between scapulae), the batch was manufactured on December 16, 2008, sent to Lanagro on December 27, 2008 and released on March 6, 2008, titration (National Institutes of Health method) was 3.06 IU/dose. Determination of serum neutralizing antibodies to rabies virus was performed at the Laboratory of Zoonoses and Vector-Borne Diseases of the Center for Zoonosis Control of São Paulo by means of the rapid fluorescent focus inhibition test (RFFIT) according to Smith *et al*. [13], modified by Zalan *et al.*[14]. A protective titer was considered when neutralizing antibodies ≥ 0.5 IU/mL [15]. In order to quantify the occurrence of protective titers, the frequency distribution of responses and the percentage of vaccine response among sampling periods were considered in relation to the initial moment, taking into account the loss of dogs during the study (207 died).

Of the 576 studied animals, none showed any evidence of protective antibody titers (<0.5 IU/mL) at moment 0. One of the biggest obstacles of this research was the loss of animals, since numerous dogs run away during the study period or were killed by cars. According to reports from the owners 207 dogs (35.9%) were lost.

Table 1 shows that 89.1% of the animals reached protective titers, indicating a growth of 12% due to the second vaccine shot. From the second moment on, the animals demonstrated a reduction in antibody titers. After 360 dafv, only 54.7% of the dogs had protective titers. The ratios between the number of protected animals and those unprotected during the study were: 8.2:1 at the first moment, 478:1 at the second moment, 7.7:1 at the third moment and 1.2:1 at the fourth moment (Table 1). Of the 576 dogs, 207 either died or run away. Then, of the remaining 369 animals, none was protected at the first moment, 27 (7.3% out of 369) were protected at moments 1 and 2, 109 (29.5%) had protective titers in the period between moments 1 and 3, 13 dogs (3.5%) were protected only in moment 2, 17 (4.6%) in moments 2 and 3, 12 dogs (3.3%) had protective titers between moments 2 and 4, one dog (0.3%) did not reach any protective level and 190 (51.5%) animals maintained protective titers during the 360 days.

**Table 1:** Percentage of dogs with protective antibody titers against rabies vaccinated with Fuenzalida-Palácios modified vaccine at four different periods, 2014

**Titer**	**30 dafv**		**60 dafv**		**180 dafv**		**360 dafv**	
	**1st moment**		**2nd moment**		**3rd moment**		**4th moment**	
	**Absolut frequency**	**Relative frequency (%)**	**Absolut frequency**	**Relative frequency (%)**	**Absolut frequency**	**Relative frequency (%)**	**Absolut frequency**	**Relative frequency (%)**
**Protected**	460	89.1	478	99.8	338	88.5	202	54.7
**Not protected**	56	10.9	1	0.2	44	11.5	167	45.3
**Total**	516	100	479	100	382	100	369	100

The low concentration of antibody titers in dogs vaccinated against rabies has been previously reported [16-19]. Similarly, the immune response of dogs vaccinated only one time has already been evaluated and the results showed a rapid drop of antibody titers, which suggests that several animals are unprotected among vaccination campaigns [10,20].

According to the Pasteur Institute [21], titers below 0.5 IU/mL do not protect animals against rabies. In the present study, 45.3% of the dogs that received the first dose followed by booster shot (30 dafv) did not present protective antibody concentrations after 12 months (Table 1), which is not an expected result.

The World Health Organization (WHO) recommends that 75% of the canine population of any country should be vaccinated. However, the program for rabies control of São Paulo state recommends a coverage of at least 80% [11,22]. In the 2009 vaccination campaign in Botucatu city, SP, the immunization coverage reached 81.36% of the animals. However, only 54.7% of the first vaccinated animals showed protective levels one year after the initial dose (Table 1).

In a study by Hirayama *et al*. [23] the same vaccine used in the current work was administered to animals without the booster doses. The authors found that the titers declined 120 dafv, therefore 40% of the animals did not have protective titers. In the present study, however, the drop of serum antibody titers (<0.5 IU/mL) in dogs occurred after the third time (180 dafv) (Table 1).

In Brazil, vaccination campaigns against rabies are annual and booster doses are recommended 30 to 45 days after the first shot. However, the booster dose is voluntary and the owner must take the animal to a health surveillance center to receive it [7]. In the present research, we found that after the third dose (180 dafv), 88.5% of the dogs remained protected against rabies virus (titer ≥ 0.5 IU/mL) (Table 1). This value was a greater value than that found by Shimazaki *et al*. [24], who evaluated the immune response in unvaccinated dogs and in animals vaccinated with a single dose of Fuenzalida- Palácios modified vaccine.

Soares *et al.*[8] observed that 120 days after vaccination, only 58% of the studied canine population, which received the vaccine for the first time, had antibody serum titers considered protective. They also reported that 30 days after the first shot, 91.2% of the dogs had protective titers whereas Almeida *et al*. [9] described only 27.4%. In the current study, at the fourth moment (360 dafv), 54.7% of the dogs had protective antibody titers, a higher value than that found by Soares *et al.*[8] who obtained 35.3% and by Almeida *et al*. [9] who found 18.3% in animals that did not receive the booster dose. When they discussed the determinants for the decline in antibody concentration after vaccination, it was hypothesized that the nutritional status, health status, and genetic ancestry may influence the immune response, the antibody induction and the maintenance of antibody titers in dogs [25].

## Conclusion

Our present findings showed that after the first vaccination, 51.5% of the dogs had protective titers during the five study periods. After a year of this first shot, only 54.7% of the animals maintained protective antibody levels against rabies. Therefore, in order to improve protective titers against rabies for 12 months, we suggest a third dose of vaccine at 180 days after the first one.

## Competing interests

The authors declare that there are no competing interests.

## Authors’ contribution

SDB, HFC, and JRM designed the study. SDB and HFC collected biological samples. LFAM and APAGK performed serology tests. CRP analyzed the data. SDB, HFC, CV and JRM drafted the manuscript, and all authors read and approved the final version of it.
